# Dihydroxy fatty acids can be used for screening autism traits in toddlers

**DOI:** 10.1002/pcn5.70338

**Published:** 2026-04-20

**Authors:** Takaharu Hirai, Naoko Umeda, Takayo Ohto‐Nakanishi, Toru Fujioka, Keisuke Wakusawa, Takahiro Nara, Kenji J. Tsuchiya, Hideo Matsuzaki

**Affiliations:** ^1^ Department of Psychiatric and Mental Health Nursing, School of Nursing University of Fukui Eiheiji Japan; ^2^ Department of Maternal and Child Health Nursing, School of Nursing University of Fukui Japan; ^3^ Lipidome Lab Co. Ltd. Akita Japan; ^4^ Department of Science of Human Development, Humanities and Social Science, Faculty of Education University of Fukui Fukui Japan; ^5^ Department of Developmental Neuropsychiatry Miyagi Children's Hospital Miyagi Japan; ^6^ Research Center for Child Mental Development Hamamatsu University School of Medicine Hamamatsu Japan; ^7^ United Graduate School of Child Development, The University of Osaka, Kanazawa University, Hamamatsu University School of Medicine Chiba University and University of Fukui Suita Japan; ^8^ Research Center for Child Mental Development University of Fukui Eiheiji Japan

Polyunsaturated fatty acids (PUFAs), such as arachidonic acid (AA), are key regulators of inflammation via their metabolism by cytochrome P450 (CYP) and soluble epoxide hydrolase.^1^, ^2^ In pregnant mice exposed to high‐dose glyphosate during gestation and lactation, offspring exhibit autism spectrum disorder (ASD)‐like behaviors accompanied by reduced AA‐derived epoxy fatty acids in plasma and multiple brain regions.^3^ AA‐derived dihydroxy fatty acids in umbilical cord blood have been associated with ASD symptoms at 6 years of age, especially in girls.^4^ As umbilical cord blood reflects the fetal period, whether abnormalities in CYP–PUFA metabolism persist into early childhood remains unclear. Given the early onset and developmental trajectory of ASD symptoms, we investigated the relationship between CYP–PUFA metabolites (epoxy fatty acids and dihydroxy fatty acids) in peripheral blood and autistic traits during early childhood.

We included 19 toddlers with ASD (mean age, 3.9 years; standard deviation [SD], 0.7) and 21 typically developing (TD) toddlers (mean age, 4.5 years; SD, 1.4). Among the 40 participants, 23 were boys, and 17 were girls (Table S1). We first examined the association between CYP–PUFA metabolites and the Autism Spectrum Quotient–Child (AQ), to identify candidates for subsequent receiver operating characteristic (ROC) analysis. A significant association was found exclusively with 14,15‐diHETrE, a dihydroxy fatty acid derived from AA (*β* = −0.368; 95% confidence interval [CI], −0.112 to −0.010; *p* = 0.021; Table S2). In contrast, no significant associations were observed with metabolites derived from linoleic acid, eicosapentaenoic acid, or docosahexaenoic acid.

We then performed sex‐stratified analyses, adjusting for the developmental quotient, which is a potential confounding factor that affects autistic traits. Figure 1a presents scatter plots and regression lines showing the relationships between AA‐derived dihydroxy fatty acids and AQ scores. Both 11,12‐ and 14,15‐diHETrE were significantly associated with autistic traits in girls (*β* = −0.455; 95% CI, −0.131 to −0.014; *p* = 0.019; *β* = −0.715; 95% CI, −0.153 to −0.031; *p* = 0.006, respectively; Table S2). Only dihydroxy fatty acids derived from AA among PUFA influenced autistic traits, specifically in girls, supporting our previous cord blood report.^4^ Notably, lower levels of these metabolites were associated with increased autistic traits. Either a decrease or an increase in AA‐derived dihydroxy fatty acids in umbilical cord blood may be related to ASD.^4^, ^5^ Such metabolic imbalances may persist beyond the fetal period and influence ASD pathophysiology via inflammatory pathways. Importantly, although AA‐derived dihydroxy fatty acids are generally pro‐inflammatory, certain subtypes can exert anti‐inflammatory effects under specific conditions.^1^, ^6^ These contrasting roles suggest that reductions in specific diHETrEs could disturb the inflammatory–anti‐inflammatory balance, potentially affecting early neurodevelopment relevant to ASD.

**Figure 1:**
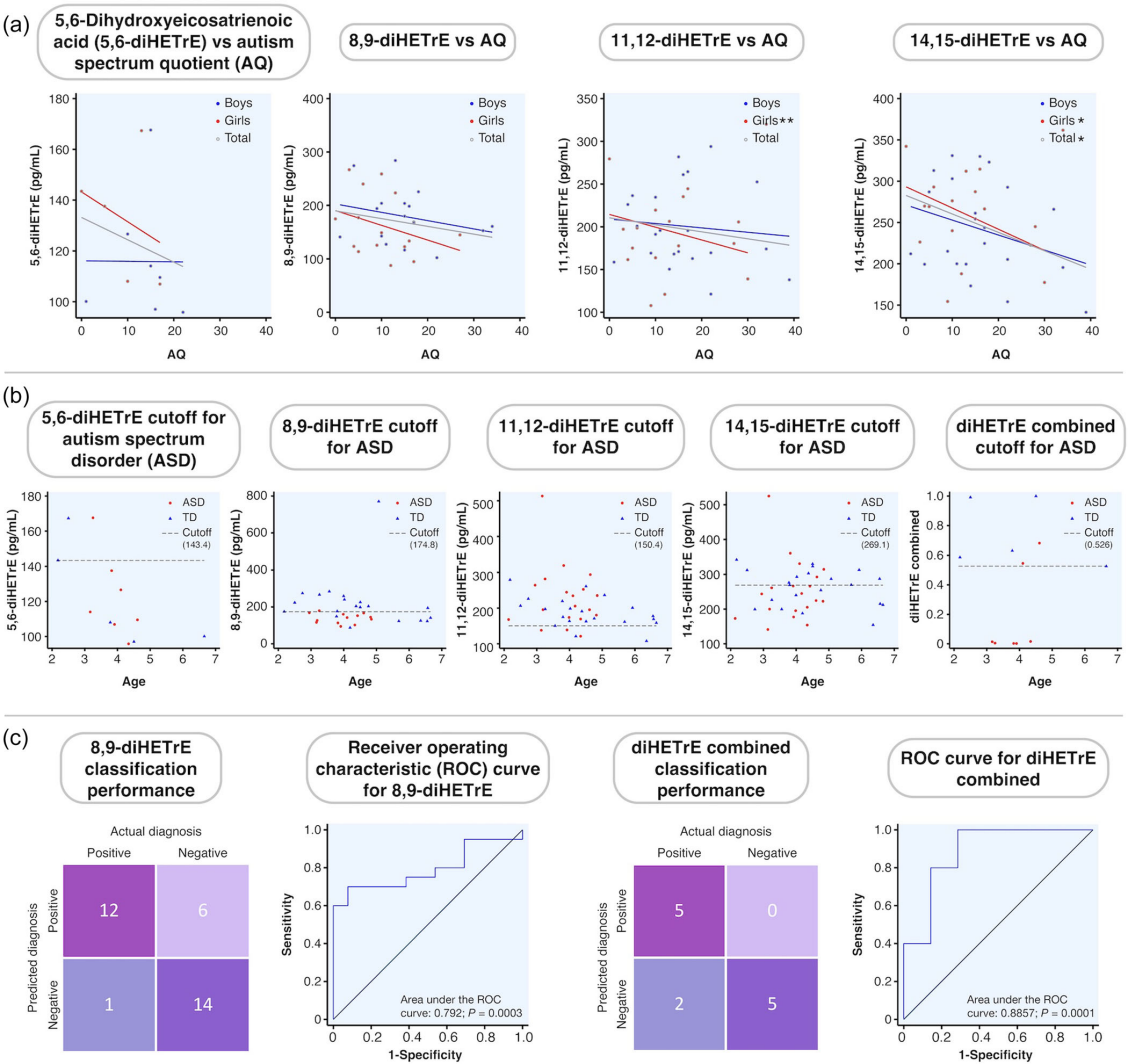
Arachidonic acid (AA)‐derived dihydroxy fatty acid derived from AA (diHETrE) as a potential biomarker for autism spectrum disorder (ASD) in early childhood. (a) Scatter plots showing the associations between Autism Spectrum Quotient–Child (AQ) scores and concentrations of four AA‐derived dihydroxy fatty acids (5,6‐, 8,9‐, 11,12‐, and 14,15‐diHETrE) based on linear regression analysis. Each plot shows data points by sex and for the total sample, with corresponding regression lines derived from the regression models. Significant negative associations with AQ scores were observed for 11,12‐diHETrE in girls and 14,15‐diHETrE in the total sample and in girls. (b) Scatter plots of diHETrE concentrations versus age, illustrating the distribution of ASD and typically developing (TD) toddlers in relation to the optimal cutoff values derived from receiver operating characteristic (ROC) analysis (indicated by horizontal dashed lines). Group separation was more pronounced at younger ages, particularly for 8,9‐diHETrE. (c) ROC curves and confusion matrices for 8,9‐diHETrE and the diHETrE combined score. Both markers demonstrated high discriminative accuracy in distinguishing ASD from TD, with 8,9‐diHETrE showing high sensitivity and the combined score achieving perfect specificity. *p < 0.05, **p < 0.01.

We next assessed the potential of AA‐derived dihydroxy fatty acids as biomarkers for ASD using ROC analysis. Among them, 8,9‐diHETrE emerged as the most promising single candidate. At an optimal cutoff point of 174.8 pg/mL, it achieved a sensitivity of 92.3%, specificity of 70%, and an AUC of 0.792 (*p* = 0.0003; Figure 1c and Table S3), outperforming other isomers. This high sensitivity captured 12 of 13 ASD cases. Although six TD toddlers were also classified as positive, this likely reflects a heightened sensitivity to early ASD‐related metabolic shifts. The discriminative pattern was particularly evident in younger participants (Figure 1b), suggesting its promise as an early screening biomarker.

We further evaluated a composite biomarker combining four AA‐derived isomers: 5,6‐, 8,9‐, 11,12‐, and 14,15‐diHETrE. The combined score achieved an AUC of 0.886 at a cutoff value of 0.526 (*p* = 0.0001; sensitivity = 71.4%, specificity = 100%; Figure 1c and Table S3). Importantly, although two of seven ASD cases were not identified, all TD toddlers were correctly classified (Figure 1c), demonstrating excellent specificity and overall classification performance. Although the small sample size warrants cautious interpretation, the clear discriminative patterns underscore the potential utility of AA‐derived diHETrEs as clinically relevant biomarkers. Given the limited number of ASD cases relative to the number of predictors, this model may be affected by overfitting, and its estimated diagnostic performance should be interpreted with caution. These findings are based on blood samples obtained during early childhood, a developmental stage in which biomarker data remain scarce due to the challenges of sample collection. Current diagnostic practices for ASD rely largely on the accumulation of behavioral features over time, which may lead to diagnostic delays or reduced consistency, particularly in early childhood.^7^ Early diagnosis is widely recognized as critical for improving long‐term outcomes in individuals with ASD because it enables timely access to appropriate interventions and support services.^8^ Moreover, interventions delivered during sensitive periods of brain development normalize neural activity patterns and improve behavioral outcomes.^9^ However, several limitations should be considered. ASD and TD toddlers were recruited from different regions in Japan, and the influence of regional factors, including dietary patterns, cannot be completely excluded, as dietary intake can modulate PUFA profiles and their downstream metabolites.^10^ Although sample collection procedures and storage conditions were standardized, slight variations in storage duration may have influenced the quantification of CYP–PUFA metabolites. In addition, although the validated age range of the Japanese AQ is 6–15 years, it was also applied in some participants younger than this range (Table S1), which should be considered while interpreting the findings. Further validation in larger‐scale studies is needed to confirm their diagnostic value and generalizability. Nonetheless, our results highlight the feasibility and promise of implementing biomarker‐based screening, even in toddlers.

## AUTHOR CONTRIBUTIONS

Hideo Matsuzaki conceived and organized this study. Takaharu Hirai analyzed the data and drafted the manuscript. Toru Fujioka, Keisuke Wakusawa, Takahiro Nara, and Kenji J. Tsuchiya provided investigation and resources. Takayo Ohto‐Nakanishi performed the LC‐MS/MS analysis. Naoko Umeda analyzed and interpreted the data. All authors contributed to the discussion of the results and the creation of this manuscript.

## CONFLICT OF INTEREST STATEMENT

T.O.‐N. is employed with Lipidome Lab Co., Ltd. The remaining authors declare that the research was conducted without any commercial or financial relationships that could be construed as a potential conflict of interest.

## ETHICS APPROVAL STATEMENT

All procedures were approved by the ethics committee of the University of Fukui and the Hamamatsu University School of Medicine, and were conducted in accordance with the Ethical Guidelines for Medical and Health Research Involving Human Subjects of the Ministry of Health, Labour and Welfare of Japan.

## PATIENT CONSENT STATEMENT

All participants were given a complete description of the study and provided written informed consent from their parent and/or legal guardian before enrollment.

## CLINICAL TRIAL REGISTRATION

N/A.

## Supporting information

Supporting Information.

## Data Availability

The data that support the findings of this study are available on request from the corresponding author. The data are not publicly available due to privacy or ethical restrictions.

## References

[1] Kundu S , Roome T , Bhattacharjee A , Carnevale KA , Yakubenko VP , Zhang R , et al. Metabolic products of soluble epoxide hydrolase are essential for monocyte chemotaxis to MCP‐1 in vitro and in vivo. J Lipid Res. 2013;54:436–447. 23160182 10.1194/jlr.M031914 PMC3588870

[2] Node K , Huo Y , Ruan X , Yang B , Spiecker M , Ley K , et al. Anti‐inflammatory properties of cytochrome P450 epoxygenase‐derived eicosanoids. Science. 1999;285:1276–1279. 10455056 10.1126/science.285.5431.1276 PMC2720027

[3] Pu Y , Yang J , Chang L , Qu Y , Wang S , Zhang K , et al. Maternal glyphosate exposure causes autism‐like behaviors in offspring through increased expression of soluble epoxide hydrolase. Proc Natl Acad Sci. 2020;117:11753–11759. 32398374 10.1073/pnas.1922287117 PMC7260984

[4] Hirai T , Umeda N , Harada T , Okumura A , Nakayasu C , Ohto‐Nakanishi T , et al. Arachidonic acid‐derived dihydroxy fatty acids in neonatal cord blood relate symptoms of autism spectrum disorders and social adaptive functioning: Hamamatsu Birth Cohort for Mothers and Children (HBC Study). Psychiatry Clin Neurosci. 2024;78:546–557. 39041066 10.1111/pcn.13710 PMC11488600

[5] Che X , Roy A , Bresnahan M , Mjaaland S , Reichborn‐Kjennerud T , Magnus P , et al. Metabolomic analysis of maternal mid‐gestation plasma and cord blood in autism spectrum disorders. Mol Psychiatry. 2023;28:2355–2369. 37037873 10.1038/s41380-023-02051-w

[6] Fang X , Hu S , Xu B , Snyder GD , Harmon S , Yao J , et al. 14,15‐Dihydroxyeicosatrienoic acid activates peroxisome proliferator‐activated receptor‐α. Am J Physiol Heart Circ Physiol. 2006;290:H55–H63. 16113065 10.1152/ajpheart.00427.2005

[7] American Psychiatric Association . Diagnostic and statistical manual of mental disorders. American Psychiatric Association; 2013.

[8] Fernell E , Eriksson MA , Gillberg C . Early diagnosis of autism and impact on prognosis: a narrative review. Clin Epidemiol. 2013;5:33–43. 23459124 10.2147/CLEP.S41714 PMC3583438

[9] Dawson G , Jones EJH , Merkle K , Venema K , Lowy R , Faja S , et al. Early behavioral intervention is associated with normalized brain activity in young children with autism. J Am Acad Child Adolesc Psychiatry. 2012;51:1150–1159. 23101741 10.1016/j.jaac.2012.08.018 PMC3607427

[10] Ostermann AI , Reutzel M , Hartung N , Franke N , Kutzner L , Schoenfeld K , et al. A diet rich in omega‐3 fatty acids enhances expression of soluble epoxide hydrolase in murine brain. Prostaglandins Other Lipid Mediat. 2017;133:79–87. 28583889 10.1016/j.prostaglandins.2017.06.001

