# Supplementary material for: Dihydroxy fatty acids can be used for screening autism traits in toddlers
Source: PCN Rep. 2026 Apr 20;5(2):e70338. doi: 10.1002/pcn5.70338 (PMC13096712; doi:10.1002/pcn5.70338)
Supplement: Supplementary file 1 — Supporting Information. [file PCN5-5-e70338-s001.docx]

**SUPPLEMENT METHODS**

**Sex as a biological variable**

Both male and female children were included. As autism spectrum disorder (ASD) exhibits known sex differences, sex was treated as a biological variable. Analyses were stratified by sex.

**Study design and participants**

This study was an observational, non-interventional investigation. Children with ASD were recruited through Miyagi Children’s Hospital (Sendai, Japan), while age-matched typically developing (TD) children were recruited via advertisements in Hamamatsu and Fukui, Japan. All participants underwent a comprehensive medical history evaluation by a physician to exclude those with neurological or medical conditions. Additionally, past and current psychiatric histories of the participants and their families were assessed using the Structured Clinical Interview for the Diagnostic and Statistical Manual of Mental Disorders, Fourth Edition (DSM-IV). Individuals diagnosed with neurological or other disorders, including fragile X syndrome, epileptic seizures, obsessive–compulsive disorder, mood disorders, schizophrenia, other psychiatric or neurological diseases, liver dysfunction, or unspecified inflammatory complications, were excluded from the study. Eligible participants had no history of pharmacological treatment, including nutritional supplements, and had not followed a cholesterol-lowering diet for at least 6 months prior to the study. All procedures involving child participants were conducted after obtaining written informed consent from the participants or their legal guardians, following a thorough explanation of the study. The procedures adhered to the protocol approved by the Ethics Committee of the University of Fukui (Approval No. #20130089) and complied with the Ethical Guidelines for Medical and Health Research Involving Human Subjects of the Ministry of Health, Labour and Welfare of Japan.

ASD was diagnosed by an experienced child psychiatrist based on the criteria outlined in the DSM-IV-Text Revision, using clinical interviews and the Japanese version of the Autism Diagnostic Interview-Revised (ADI-R).^1^ The ADI-R was administered by one of the study authors (TF), who had established research reliability in collaboration with the developers of the ADI-R or independent trainers. The ADI-R provides an autism diagnostic algorithm aligned with the International Classification of Diseases, 10th Revision, and the DSM-IV. It is a semi-structured psychiatric interview primarily conducted with mothers. While ASD diagnosis was based on categorical criteria using the ADI-R and DSM-IV-TR, the study also aimed to capture dimensional variation in autistic traits across the broader population. To assess autistic traits, the Autism Spectrum Quotient-Child (AQ) was used as the primary outcome.^2^ The AQ consists of 50 items evaluating ASD-related characteristics, with parents rating each item on a four-point Likert scale: definitely agree, slightly agree, slightly disagree, and definitely disagree. The AQ includes domains assessing attention switching, attention to detail, communication, social skills, and imagination. In this study, the total AQ score, calculated by summing the five subscale scores, was used as a continuous measure of autistic traits, with higher scores indicating a greater degree of autistic traits. The AQ allows identification of individuals in the “gray zone” who do not exhibit clear clinical symptoms but display ASD-related traits from a dimensional perspective, providing a nuanced understanding of autism as a spectrum disorder. The Japanese version of the AQ was used in this study.^3,4^ Although the validated age range of the Japanese AQ is 6–15 years, it was also applied to some participants younger than this range in the present study.

To assess participants’ cognitive development, the Kyoto Scale of Psychological Development (KSPD) was used to evaluate the developmental quotient (DQ). The KSPD is one of the most widely used developmental assessments in Japan, and DQ evaluation using the KSPD is considered an alternative to intelligence quotient assessment.^5^ This equivalence has also been recognized in young children with ASD.^6^ The KSPD is an individually administered, face-to-face assessment conducted by experienced psychologists. The total score is converted into a developmental age (DA), and the DQ is calculated as follows: (DA/chronological age) × 100.

**Blood sampling and fatty acid analysis**

Fasting blood samples were collected from all participants between 7:00 AM and noon while they were in a seated position, using venipuncture with a tourniquet. After standing at room temperature for 30 min, the samples were centrifuged at 3,500 × *g* for 10 min at 4°C. The supernatant was collected as serum, aliquoted into 200 μL portions, and stored at −80°C until required.

Liquid chromatography–mass spectrometry (LC-MS/MS) analysis was performed to quantify cytochrome P450 (CYP)–polyunsaturated fatty acid (PUFA) metabolites in the serum. Specifically, the lipid fraction containing epoxide fatty acids was extracted from 180 μL of serum using Oasis HLB solid-phase extraction columns (Waters Corporation, Milford, MA, USA). Epoxide fatty acids were further separated using a high-performance liquid chromatography system (Nexera LC-30AD, Shimadzu Corporation, Kyoto, Japan) equipped with an XBridge C18 column (particle size, 3.5 μm; length, 150 mm; inner diameter, 1.0 mm; Waters) and analyzed on a triple quadrupole mass spectrometer (LC-MS-8040; Shimadzu). Mass spectrometric analysis was conducted in negative-ion mode, and fatty acid metabolites were identified and quantified by multiple-reaction monitoring, consistent with the determination of other lipid metabolites.^7^

For quantification, calibration curves were prepared for each compound, and recoveries were monitored using deuterated internal standards (11,12-epoxy eicosatrienoic acid-d11, 12,13-dihydroxy octadecenoic acid-d4, and arachidonic acid-d8; Cayman Chemicals, Ann Arbor, MI, USA). Data were analyzed using LabSolutions software (Shimadzu). LC-MS/MS analysis was performed according to the protocols of Lipidome Lab Co., Ltd. Values below the limit of detection were excluded from the analysis, whereas those below the limit of quantitation were included.

**Statistical analysis**

To identify candidate CYP-PUFA metabolites associated with autistic traits, we first conducted linear regression analyses using AQ total scores as the dependent variable and each metabolite as an independent variable. Based on our previous findings on sex differences,^8^ sex-stratified multivariable linear regression analyses were also performed, adjusting for potential confounders, such as DQ.^5^ Outliers were excluded using the interquartile range method prior to regression analysis to ensure robust model estimation.

Candidate metabolites identified from these regression analyses were subsequently subjected to receiver operating characteristic (ROC) analysis to evaluate their ability to discriminate between ASD and TD toddlers. For ROC analysis, the full dataset—including outliers—was used to better reflect real-world classification performance. For each metabolite, sensitivity, specificity, positive likelihood ratio, correct classification rate, and area under the curve (AUC) were calculated, and the optimal cutoff value was determined using Youden’s index.

Additionally, to assess the predictive ability of models combining multiple variables, a predictive score was calculated from a logistic regression model as an integrated score and applied to ROC analysis. All statistical analyses were performed using Stata version 18.0 (StataCorp LLC, College Station, TX, USA), with a significance level set at *P* <0.05.

**REFERENCES**

1. Tsuchiya KJ, Matsumoto K, Yagi A, et al. Reliability and validity of autism diagnostic interview-revised, Japanese version. *J Autism Dev Disord* 2013; **43:** 643–62.

2. Baron-Cohen S, Wheelwright S, Skinner R, et al. The autism-spectrum quotient (AQ): evidence from Asperger syndrome/high-functioning autism, males and females, scientists and mathematicians. *J Autism Dev Disord* 2001; **31:** 5–17.

3. Wakabayashi A, Baron-Cohen S, Wheelwright S, Tojo Y. The Autism-Spectrum Quotient (AQ) in Japan: a cross-cultural comparison. *J Autism Dev Disord* 2006; **36:** 263–70.

4. Wakabayashi A, Uchiyama T, Tojo Y, et al. Autism-spectrum quotient (AQ) Japanese children's version " comparison between high-functioning children with autism spectrum disorders and normal controls. *Shinrigaku Kenkyu* 2007; **77:** 534–40 (in Japanese).

5. Kawabe K, Kondo S, Matsumoto M, et al. Developmental quotient to estimate intelligence in autism spectrum disorder. *Pediatr Int* 2016; **58:** 963–6.

6. Koyama T, Osada H, Tsujii H, Kurita H. Utility of the Kyoto Scale of Psychological Development in cognitive assessment of children with pervasive developmental disorders. *Psychiatry Clin Neurosci* 2009; **63:** 241–3.

7. Hijioka M, Futokoro R, Ohto-Nakanishi T, Nakanishi H, Katsuki H, Kitamura Y. Microglia-released leukotriene B4 promotes neutrophil infiltration and microglial activation following intracerebral hemorrhage. *Int Immunopharmacol* 2020; **85:** 106678.

8. Hirai T, Umeda N, Harada, T, et al. Arachidonic acid-derived dihydroxy fatty acids in neonatal cord blood relate symptoms of autism spectrum disorders and social adaptive functioning: Hamamatsu Birth Cohort for Mothers and Children (HBC Study). *Psychiatry Clin Neurosci* 2024; **78:** 546–57.

**SUPPLEMENTAL TABLES**

**Supplemental Table 1. Characteristics of toddlers**





**Supplemental** **Table 2. CYP-PUFA metabolites associated with autistic traits in total and by sex**





**Supplemental** **Table 3. Discriminative performance of dihydroxy fatty acids for ASD screening**
